# A 39-Amino-Acid C-Terminal Truncation of GDV1 Disrupts Sexual Commitment in Plasmodium falciparum

**DOI:** 10.1128/mSphere.01093-20

**Published:** 2021-05-19

**Authors:** Marta Tibúrcio, Eva Hitz, Igor Niederwieser, Gavin Kelly, Heledd Davies, Christian Doerig, Oliver Billker, Till S. Voss, Moritz Treeck

**Affiliations:** a Signalling in Apicomplexan Parasites Laboratory, The Francis Crick Institute, London, United Kingdom; b Department of Medical Parasitology and Infection Biology, Swiss Tropical and Public Health Institute, Basel, Switzerland; c University of Basel, Basel, Switzerland; d Bioinformatics Science Technology Platform, The Francis Crick Institute, London, United Kingdom; e Billker Group, Rodent Models of Malaria, Wellcome Sanger Institute, Wellcome Genome Campus, Hinxton, Cambridge, United Kingdom; f Centre for Chronic Infectious and Inflammation Disease, Biomedical Sciences Cluster, School of Health and Biomedical Sciences, RMIT University, Bundoora, Australia; g Department of Molecular Biology and Molecular Infection Medicine Sweden, Umeå, Sweden; The Hebrew University of Jerusalem

**Keywords:** GDV1, gametocytes, *Plasmodium falciparum*, transmission, kinases

## Abstract

Malaria is a mosquito-borne disease caused by apicomplexan parasites of the genus *Plasmodium.* Completion of the parasite’s life cycle depends on the transmission of sexual stages, the gametocytes, from an infected human host to the mosquito vector. Sexual commitment occurs in only a small fraction of asexual blood-stage parasites and is initiated by external cues. The gametocyte development protein 1 (GDV1) has been described as a key facilitator to trigger sexual commitment. GDV1 interacts with the silencing factor heterochromatin protein 1 (HP1), leading to its dissociation from heterochromatic DNA at the genomic locus encoding AP2-G, the master transcription factor of gametocytogenesis. How this process is regulated is not known. In this study, we have addressed the role of protein kinases implicated in gametocyte development. From a pool of available protein kinase knockout (KO) lines, we identified two kinase knockout lines which fail to produce gametocytes. However, independent genetic verification revealed that both kinases are not required for gametocytogenesis but that both lines harbor the same mutation that leads to a truncation in the extreme C terminus of GDV1. Introduction of the identified nonsense mutation into the genome of wild-type parasite lines replicates the observed phenotype. Using a GDV1 overexpression line, we show that the truncation in the GDV1 C terminus does not interfere with the nuclear import of GDV1 or its interaction with HP1 *in vitro* but appears to be important to sustain GDV1 protein levels and thereby sexual commitment.

**IMPORTANCE** Transmission of malaria-causing *Plasmodium* species by mosquitos requires the parasite to change from a continuously growing asexual parasite form growing in the blood to a sexually differentiated form, the gametocyte. Only a small subset of asexual parasites differentiates into gametocytes that are taken up by the mosquito. Transmission represents a bottleneck in the life cycle of the parasite, so a molecular understanding of the events that lead to stage conversion may identify novel intervention points. Here, we screened a subset of kinases we hypothesized to play a role in this process. While we did not identify kinases required for sexual conversion, we identified a mutation in the C terminus of the gametocyte development 1 protein (GDV1), which abrogates sexual development. The mutation destabilizes the protein but not its interaction with its cognate binding partner HP1. This suggests an important role for the GDV1 C terminus beyond trafficking and protein stability.

## INTRODUCTION

Malaria is a devastating disease caused by parasites of the genus *Plasmodium*, leading to ∼405,000 deaths per year (1). Plasmodium falciparum causes the most severe and life-threatening form of human malaria. The complex life cycle involves interactions with multiple tissues in two different organisms, the human host and the mosquito vector. Inside the human host, P. falciparum predominantly infects red blood cells (RBC) where it asexually replicates. A small fraction (0 to 20%) of parasites commits to sexual development (gametocytogenesis) (2). Gametocytogenesis occurs preferentially in the extravascular compartment in the bone marrow and spleen (3–9). After 10 to 12 days, mature stage V gametocytes are released into the peripheral circulation to allow transmission to mosquitoes.

Sexual commitment can be initiated by metabolic cues in the human host. Specifically, it has been described that depletion of lysophosphatidylcholine (LysoPC), a common component of human serum, leads to increased rates of gametocyte production and therefore represents the first molecularly defined factor known to inhibit or trigger sexual conversion (10). Sexual commitment depends on upregulation of the *ap2-g* gene (2, 11), which requires removal of heterochromatin protein 1 (HP1) from chromatin. HP1 interacts directly with the gametocyte development 1 protein (GDV1), which causes HP1 to dissociate (12). HP1 is responsible for repression of a range of genes (13), while GDV1 specifically acts on the *ap2-g* locus. How this specificity is achieved is not known. Furthermore, how a drop in LysoPC levels is sensed and transduced into GDV1-mediated HP1 removal is not understood.

Kinases are key transducers of signals in cellular processes in various stages of the *Plasmodium* life cycle (14, 15) and are likely candidates to play important roles in gametocyte commitment and development. A study by Solyakov et al. (14) has identified a panel of likely and confirmed nonessential protein kinases, some of which are transcribed during sexual development (PlasmoDB) or in gametocytes (16–19). Aiming to identify protein kinases involved in sexual development, we screened eight knockout (KO) lines for phenotypes in gametocyte induction and/or maturation. Two lines made no gametocytes, but subsequent validation showed that their gametocytogenesis defect was not due to the absence of these kinases. Instead, we found that both lines shared the same truncation in the C-terminal end of GDV1, which caused the loss of gametocyte development. Here, we address the importance and role of the GDV1 C-terminal for sexual commitment and interaction with HP1. We show that the loss of the C-terminal 39 amino acids of GDV1 does not interfere with nuclear import and interaction with HP1 *in vitro* but prevents GDV1 from triggering efficient sexual commitment.

## RESULTS

### Identification and characterization of Plasmodium falciparum kinase KO lines with a gametocytogenesis phenotype.

**FIG 1:**
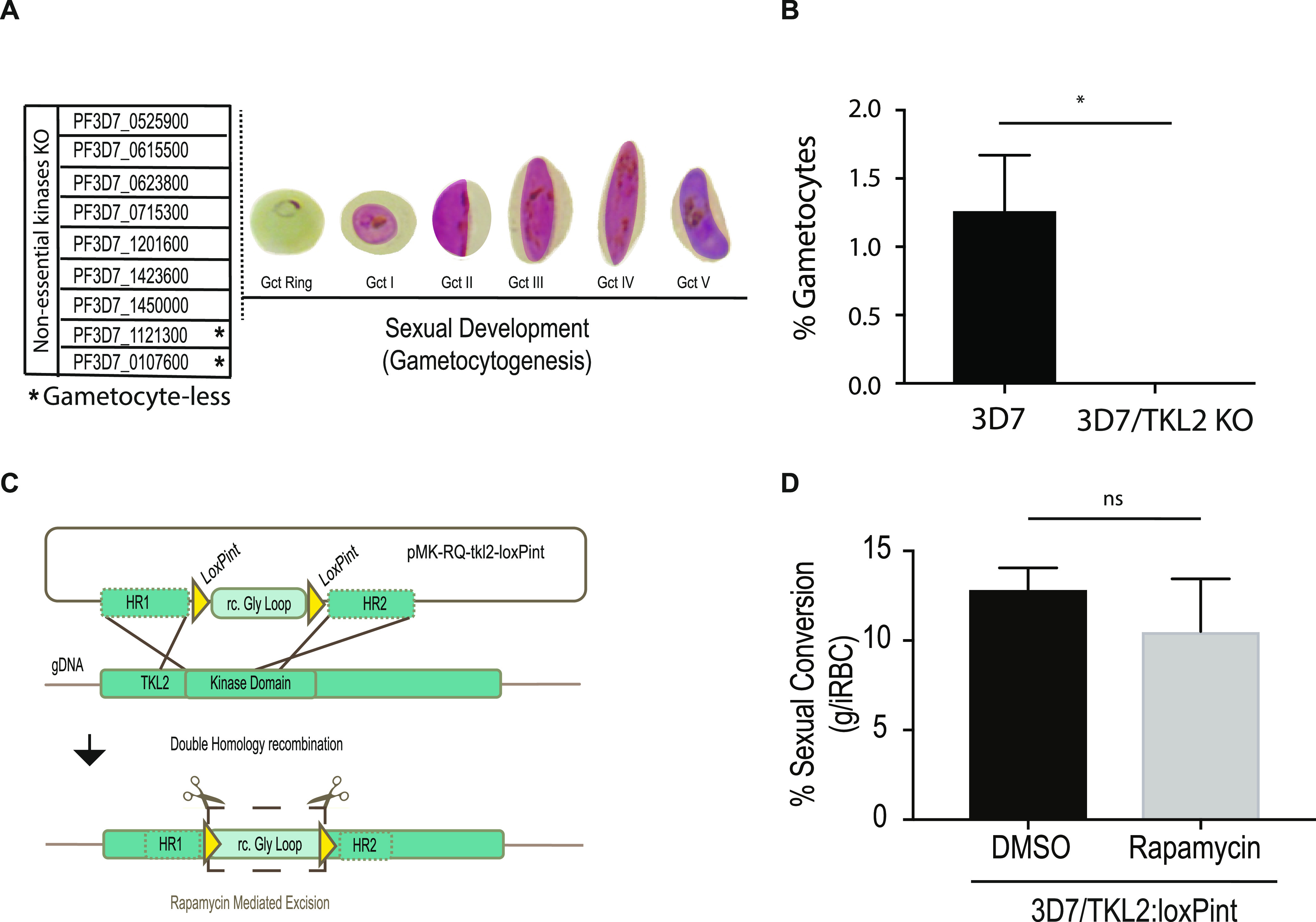
Screening of Plasmodium falciparum nonessential kinases during sexual commitment and development. (A) List of nonessential kinases characterized during sexual development in this study (PF3D7_0107600, eIK2; PF3D7_0525900, NEK2; PF3D7_0615500, CRK5; PF3D7_0623800, TKL4; PF3D7_0715300, calcium/calmodulin-dependent protein kinase, putative; PF3D7_1121300, TKL2; PF3D7_1201600, NEK3; PF3D7_1423600, calcium-dependent protein kinase, putative; PF3D7_1450000, serine/threonine protein kinase, putative) (14). (B) Comparison of the percentage of gametocytemia between the 3D7 WT line and the PfTKL2 kinase KO clones of the same transfection (clones B10 and B12) generated by single crossover integration (14). Each column represents the mean of triplicate microscope counts, each of at least 500 cells, analyzed using paired *t* test, ± standard deviation (*, *P* < 0.05; 3D7 versus TKL2 KO clones, *P* = 0.0377). (C) Schematic of the CRISPR/Cas9 strategy used to generate a TKL2 conditional knockout (KO) line (3D7/TKL2:loxPint) as well as the primers used to confirm successful gene editing (Table S2). The *pMK-RQ-tkl2-loxPint* donor plasmid contains a recodonized version of the glycine loop in the kinase domains of *tkl2* (rc. Gly oop) flanked by two loxPints and homology regions for homology-directed repair. (D) Sexual conversion rates in 3D7/TKL2:loxPint parasites treated with DMSO (control) or rapamycin (KO). Each column represents the mean of triplicate microscope counts, each of at least 500 cells, analyzed using paired *t* test ± SD, (ns, *P* ≥ 0.05; 3D7/TKL2:loxPint treated with DMSO versus rapamycin, *P* = 0.4017).

It has been shown previously in P. berghei that protein kinases that are nonessential during the asexual blood stages are essential in other life cycle stages, for example, during parasite transmission in the mosquito (15). To identify kinases important for gametocytogenesis, we investigated the role of a group of likely nonessential kinases (14) during asexual blood stages development. Using the lines described by Solyakov et al. (14), which have been generated by single crossover gene disruption, we induced sexual development using conditioned medium (20) and followed progression through stages I to V of gametocytogenesis (Fig. 1A). Six of the eight KO lines displayed normal gametocyte development, while two, TKL2 (PF3D7_1121300) and eIK2 (PF3D7_0107600) kinase KO lines, produced very few (≤0.1%) gametocytes (Fig. 1B). Of these, one has a disrupted tyrosine kinase-like 2 (*tkl2*) locus, which has been characterized as a protein kinase secreted outside the red blood cell (17). Gene loss and accumulation of mutations are frequently observed in parasite lines kept in continuous *in vitro* culture over time, and the loss of the ability to form gametocytes is not uncommon (21). To exclude mutations in the *ap2-g* gene, which was identified previously through a loss-of-function mutant (2), we sequenced the *ap2-g* locus in the 3D7/TKL2 KO parasite line. The sequencing results confirmed that the phenotype observed was not associated with mutations in *ap2-g*, leading us to conclude that the deletion of TKL2 was possibly the cause for the observed phenotype. In order to verify the role of TKL2 in gametocyte induction, we generated a DiCre-mediated TKL2 conditional KO line in NF54 parasites (NF54/TKL2:loxPint). We used CRISPR/Cas9 to simultaneously introduce a DiCre cassette into the *pfs47* locus, as previously described (22, 23), and to flank the kinase domain of *tkl2* with two loxPints (Fig. 1C and Fig. S1A and B). To address the role of TKL2 in gametocyte development, we treated the NF54/TKL2:loxPint line with dimethyl sulfoxide (DMSO; control) or rapamycin (KO) (Fig. S1B). We then induced sexual commitment using conditioned medium (20) and monitored gametocyte development. No difference in commitment or development between the control and the rapamycin-induced NF54/TKL2:loxPint parasites (Fig. 1D) was observed. These results show that TKL2 is not involved in sexual commitment or gametocyte development/maturation and that another mutation is likely the cause for the observed phenotype.

10.1128/mSphere.01093-20.1 FIG S1 Confirmation of *tkl2-loxPint* cassette integration into the Plasmodium falciparum 3D7 line and the efficiency of DiCre mediated excision. (A) Representation of the primer pairs used to test correct integration of *tkl2-loxPint* cassette and efficient rapamycin mediated excision. (B) PCR analysis shows correct integration of *tkl2-loxPint* cassette and near complete excision of TKL2:LoxPint in two different clones from the same transfection (clone 13 and 33) after rapamycin treatment. The sequences of the primers used are in Table S2. Download FIG S1, JPG file, 0.2 MB. Copyright © 2021 Tibúrcio et al. 2021 Tibúrcio et al. https://creativecommons.org/licenses/by/4.0/ This content is distributed under the terms of the Creative Commons Attribution 4.0 International license.

10.1128/mSphere.01093-20.6 TABLE S2 Primers, fragment, and guide RNA sequences used in this study. Download Table S2, DOCX file, 0.02 MB. Copyright © 2021 Tibúrcio et al. 2021 Tibúrcio et al. https://creativecommons.org/licenses/by/4.0/ This content is distributed under the terms of the Creative Commons Attribution 4.0 International license.

### A common GDV1 truncation is found in both kinase KO lines deficient in gametocyte formation.

The second kinase KO where a gametocytogenesis defect was identified was the eukaryotic initiation factor serine/threonine kinase 2 (eIK2) KO line (3D7/eIK2 KO) (Fig. S2A). eIK2 has previously been characterized as nonessential during sexual development in P. falciparum and P. berghei, and elK2 KO lines appeared to undergo normal gametocyte development in rodent *Plasmodium* species (24). This indicated that, as does 3D7/TKL2 KO, the 3D7/eIK2 KO line also harbors a mutation preventing efficient gametocyte development. Sequencing of the *ap2-g* locus in this parasite line as described above showed no mutations in the coding region of *ap2-g*. Therefore, a potentially unknown mutation underlies the loss of gametocytes in these parasite lines.

10.1128/mSphere.01093-20.2 FIG S2 PfeIK2 kinase KO parasites fail to produce gametocytes. (A) Comparison of gametocytemia upon sexual induction between the 3D7 WT line (*n* = 4) and two PfeIK2 kinase KO clones (*n* = 2 for each clone, both from the same transfection). Parasite line and clones were measured in at least two biological experiments with single replicates. Each column represents the mean number of gametocytes in at least 500 cells. (B) Representation of GDV1 sequences of the NF54, 3D7, PfeiK2, and TKL2 parasite lines and the identified point mutation in the PfeIK2 and PfTKL2 KO clones (red arrow) which is absent in the 3D7 and NF54 reference parasite lines. Download FIG S2, JPG file, 0.4 MB. Copyright © 2021 Tibúrcio et al. 2021 Tibúrcio et al. https://creativecommons.org/licenses/by/4.0/ This content is distributed under the terms of the Creative Commons Attribution 4.0 International license.

**FIG 2:**
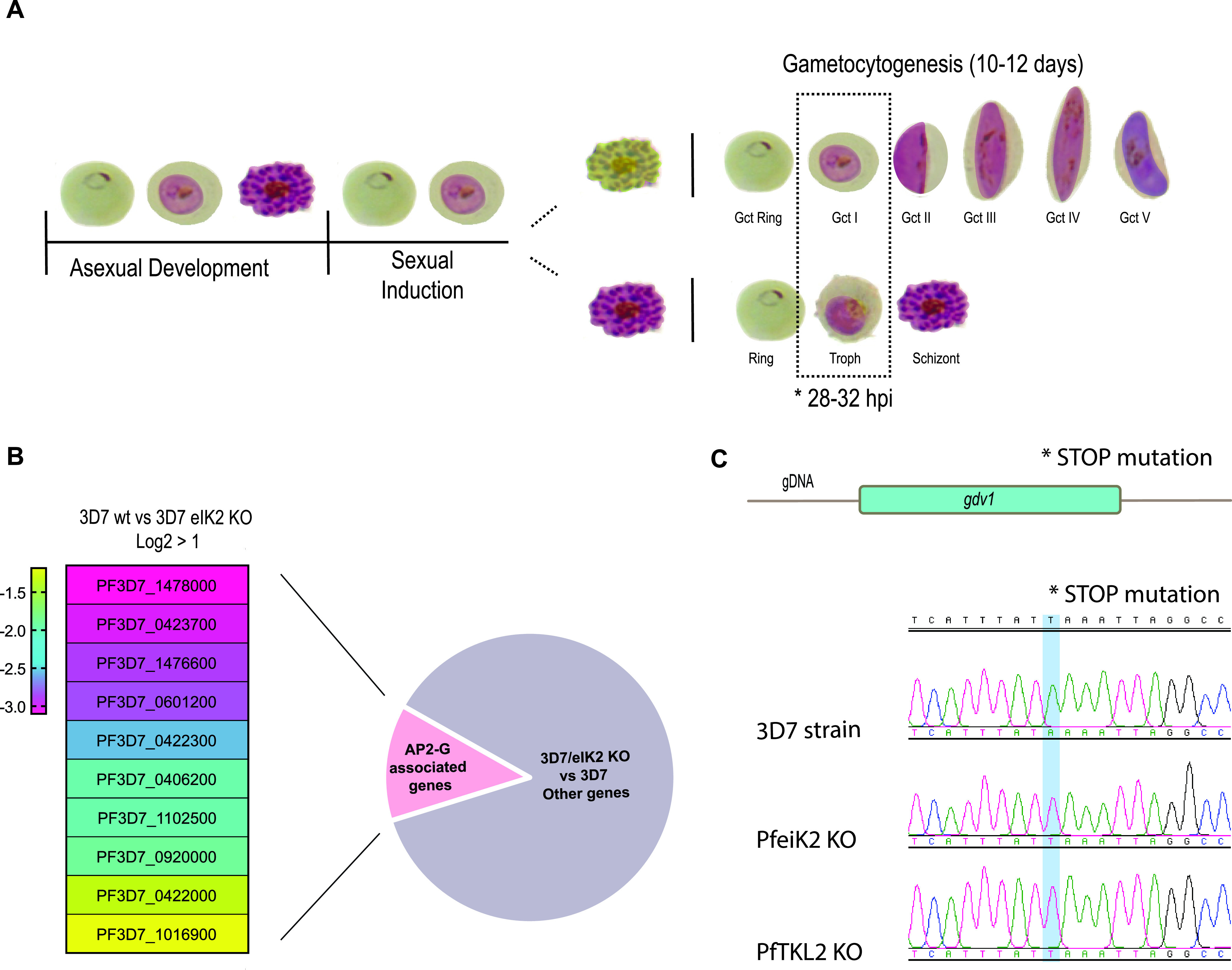
RNA sequencing analysis comparing WT and PfeIK2 gametocyte-less kinase KO line. (A) Representation of parasite differentiation upon sexual induction; dotted box illustrates the time point and asexual and sexual stages of the parasite collected for RNA-seq. (B) Heatmap showing some genes previously described as being associated with sexual commitment and early sexual differentiation (2) that are significantly downregulated in the PfeIK2 kinase KO clones (log_2_ fold change > 1). (C) DNA sequence trace showing the stop mutation identified in the PfeIK2 and PfTKL2 KO clones which is absent in the 3D7 reference parasite line.

To understand the nature of the block in sexual development, we analyzed the transcriptome of induced wild-type 3D7 parasites and two eIK2 KO clones (clones C3 and F12) using transcriptome sequencing (RNA-seq). Samples were collected for RNA extraction between 28 and 32 h postinvasion (hpi) after induction with conditioned medium (Fig. 2A). The RNA-seq analysis revealed a significant downregulation in 3D7/eIK2 KO parasites of genes known to be upregulated during gametocytogenesis, including genes that have been shown to be AP2-G-dependent (2, 10, 12, 25–27) (Fig. 2B and Table S1). We found *ap2-g* itself to be downregulated in 3D7/eIK2 KO parasites, but this reached significance only in one of the clones. Together with the lack of mutations in *ap2-g* itself, these results suggested that the block in gametocytogenesis was upstream of AP2-G function during sexual commitment. At that time, GDV1 was shown to be an upstream activator of AP2-G expression (12), so we sequenced the *gdv1* locus in the elK KO clones and identified a nonsense mutation in *gdv1* that results in a premature stop codon leading to a C-terminal truncation of 39 amino acids (GDV1Δ39) (Fig. 2C and Fig. S2B). Sequencing of the 3D7/TKL2 KO parasite clones showed the same mutation (Fig. S2B), suggesting that the deletion of the last 39 amino acids of GDV1 in both mutant lines is responsible for the gametocytogenesis phenotype observed in both kinase KO lines.

10.1128/mSphere.01093-20.5 TABLE S1 Comparison of gene expression by RNA-seq in uninduced and induced 3D7 WT and PfeIK2 kinase KO clones C3 and F12. Normalized, averaged readcounts and log_2_ fold changes of uninduced (ui) and induced (i) 3D7 and *gdv1* mutant parasite clones C3 and F12. Shortlist of genes identified as less expressed in GDV1 mutants compared to those in parental parasites and comparison to datasets from Kafsack et al. 2014 (2) and Filarsky et al. 2018 (12). Download Table S1, XLSX file, 1.9 MB. Copyright © 2021 Tibúrcio et al. 2021 Tibúrcio et al. https://creativecommons.org/licenses/by/4.0/ This content is distributed under the terms of the Creative Commons Attribution 4.0 International license.

### The carboxy-terminal 39 amino acids of GDV1 are important for its function.

**FIG 3:**
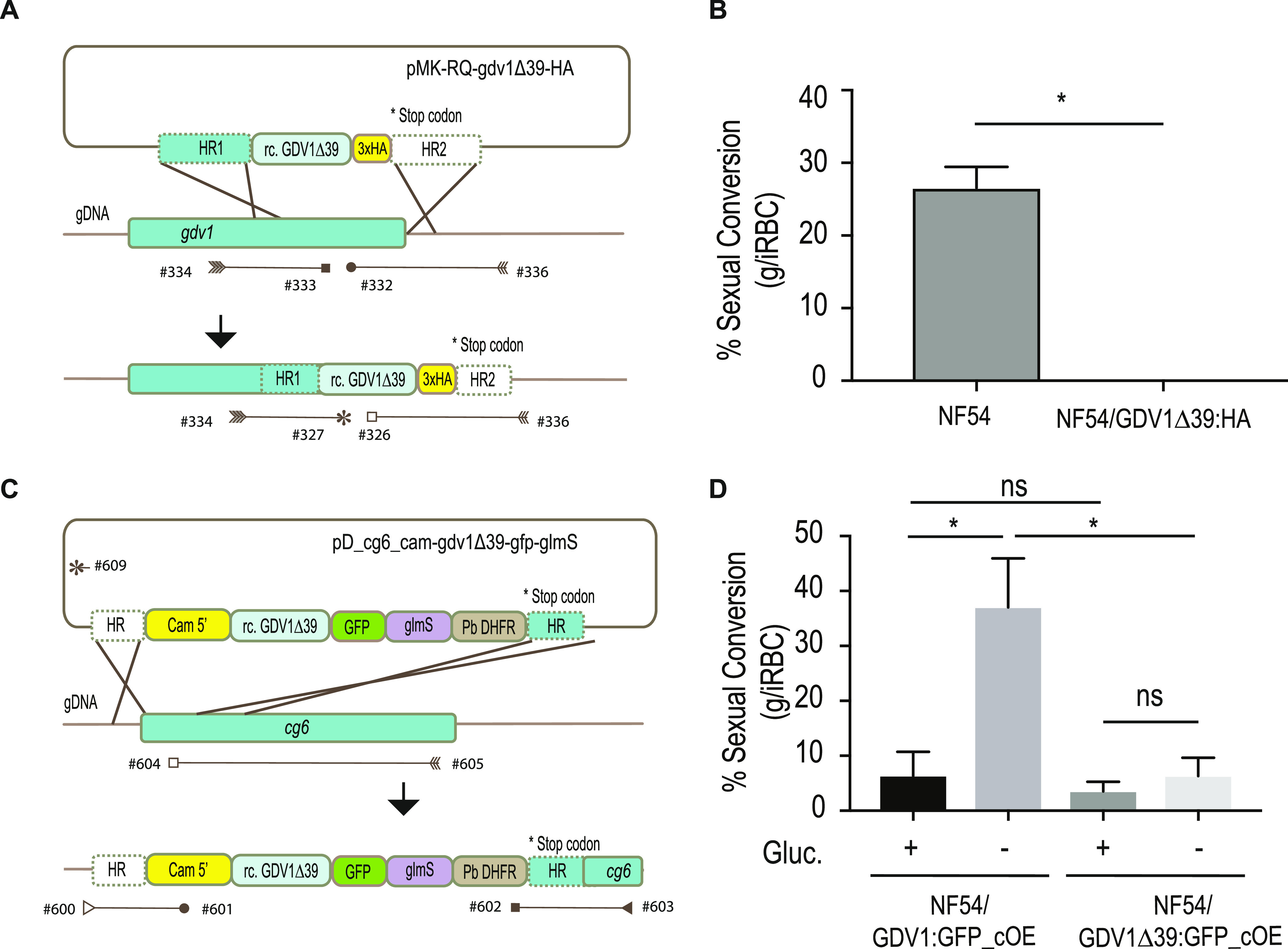
Quantification of gametocyte production in GDV1Δ39 mutant parasite lines. (A) Illustration of the strategy used to generate the 3×HA-tagged GDV1Δ39 mutant line (NF54/GDV1Δ39:HA) as well as the primers used to confirm integration (Table S2). The *pMK-RQ-gdv1Δ39-HA* donor plasmid contains a recodonized version of the *gdv1Δ39* mutant and a 3×HA tag, flanked by homology regions. (B) Comparison of sexual conversion rates between NF54 and NF54/GDV1Δ39:HA parasite lines. Each column represents the mean of duplicate (NF54) and triplicate (NF54/GDV1Δ39:HA) microscope counts, each of at least 500 cells, analyzed using paired *t* test, ± SD, (*, *P* < 0.05; NF54 versus NF54/GDV1Δ39:HA, *P* = 0.0489). (C) Schematic of the strategy used to make the NF54/GDV1Δ39:GFP_cOE overexpressing line as well as the primers used to verify integration of the transgene cassette into the *cg6* (*glp3*) locus (Table S2). The *pD_cg6_cam-gdv1Δ39-gfp-glmS* donor plasmid contains a recodonized version of the *gdv1Δ39* mutant followed by the in-frame *gfp* sequence and the *glmS* ribozyme element, flanked by homology regions. (D) Comparison of sexual conversion rates between NF54/GDV1:GFP_cOE and NF54/GDV1Δ39:GFP_cOE parasite lines in the presence (prevents sexual conversion) or absence of glucosamine (induces sexual conversion). Each column represents the mean of triplicate counts of at least 500 cells, analyzed using paired *t* test, ± SD, (*, *P* < 0.05; ns, not significant, *P* ≥ 0.05; NF54/GDV1:GFP_cOE noninduced versus induced, *P* = 0.0094; noninduced NF54/GDV1:GFP_cOE versus NF54/GDV1Δ39:GFP_cOE, *P* = 0.2276; NF54/GDV1Δ39:GFP_cOE noninduced versus induced, *P* = 0.4038; induced NF54/GDV1:GFP_cOE versus NF54/GDV1Δ39:GFP_cOE, *P* = 0.0281).

To verify genetically the identified mutation in *gdv1*, we generated a 3× hemagglutinin (3×HA)-tagged version of GDV1Δ39 and introduced it in the endogenous *gdv1* locus in the NF54 parasite line (NF54/GDV1Δ39:HA) (Fig. 3A and Fig. S3A and B). GDV1Δ39:HA parasites lost the ability to form gametocytes (Fig. 3B), suggesting that the GDV1 C terminus plays an essential role during sexual commitment or development. Determination of the localization or expression levels of GDV1Δ39:HA was not possible, as we could not confidently distinguish true signal from background fluorescence. We repeatedly failed to obtain parasites expressing 3×HA-tagged full-length GDV1 from the endogenous locus to compare its expression levels and the localization to those of the truncated GDV1 version. Notably, direct C-terminal tagging of GDV1 at the endogenous locus was also not successful in other studies, unless when in combination with a destabilization domain (12, 28).

10.1128/mSphere.01093-20.3 FIG S3 Generation of the NF54/GDV1Δ39:HA and NF54/GDV1Δ39:GFP_cOE Plasmodium falciparum lines. (A) Illustration of the strategy used to generate the 3×HA-tagged GDV1Δ39 mutant line (NF54/GDV1Δ39:HA) as well as the primers used to confirm donor sequence integration. (B) PCR analysis of integration of the gdv1Δ39:ha construct into the *gdv1* locus in the NF54 P. falciparum parasite line. (C) Schematic of the strategy used to generate the NF54/GDV1Δ39:GFP_cOE overexpressing line as well as the primers used to verify integration of the transgene cassette into the *cg6 (glp3*) locus. The pGDV1Δ39:GFP_cOE donor plasmid contains a 5′ Cam sequence followed by a recodonized version of the *gdv1Δ39* mutant in-frame with *gfp* sequence, the *glmS* ribozyme element, and Plasmodium berghei dihydrofolate reductase (*Pf*DHFR), flanked by two homology regions. (D) PCR analysis of *gdv1Δ39-gfp-glmS* cassette integration in the cg6 locus in the NF54 P. falciparum parasite line and the presence of the CRISPR/Cas9/Suicide plasmid. (E) Quantification of GFP expression in the GDV1:GFP and GDV1Δ39:GFP lines cultured in the presence (prevents GDV1 expression) or absence of glucosamine (induces GDV1 overexpression). Download FIG S3, JPG file, 1 MB. Copyright © 2021 Tibúrcio et al. 2021 Tibúrcio et al. https://creativecommons.org/licenses/by/4.0/ This content is distributed under the terms of the Creative Commons Attribution 4.0 International license.

Therefore, we resorted to a system that allows robust testing of GDV1-dependent gametocyte induction by conditional overexpression of GDV1-green fluorescent protein (GFP) and quantifying sexual conversion (29). To do that, we first introduced an ectopic *gdv1-gfp* fusion gene under the control of the calmodulin promoter and a *glmS* ribozyme in the 3′ untranslated region into the *cg6* (*glp3*, PF3D7_0709200) locus in NF54 parasites. In the presence of glucosamine, the *glmS* ribozyme destabilizes the mRNA preventing GDV1:GFP expression, while in the absence of glucosamine GDV1:GFP is overexpressed, leading to gametocyte induction (30). For simplicity, the NF54/iGP2 line described by Boltryk and colleagues (29) has been renamed NF54/GDV1:GFP_cOE in this study (cOE stands for conditional overexpression).

To test GDV1Δ39 function, we introduced a *gdv1Δ39-gfp-glmS* cassette into the *cg6* locus, generating a conditional GDV1Δ39:GFP overexpression parasite line (NF54/GDV1Δ39:GFP_cOE) (Fig. 3D and Fig. S3C and D). We then compared the sexual conversion rates in the NF54/GDV1:GFP_cOE and NF54/GDV1Δ39:GFP_cOE parasites in the presence and absence of glucosamine. In contrast to that of GDV1:GFP, overexpression of GDV1Δ39:GFP failed to trigger a significant increase of sexual commitment (Fig. 3D). These results suggest that the full integrity of the GDV1 C terminus is important for sexual commitment.

### GDV1Δ39 is imported into the nucleus and retains the ability to interact with HP1.

**FIG 4:**
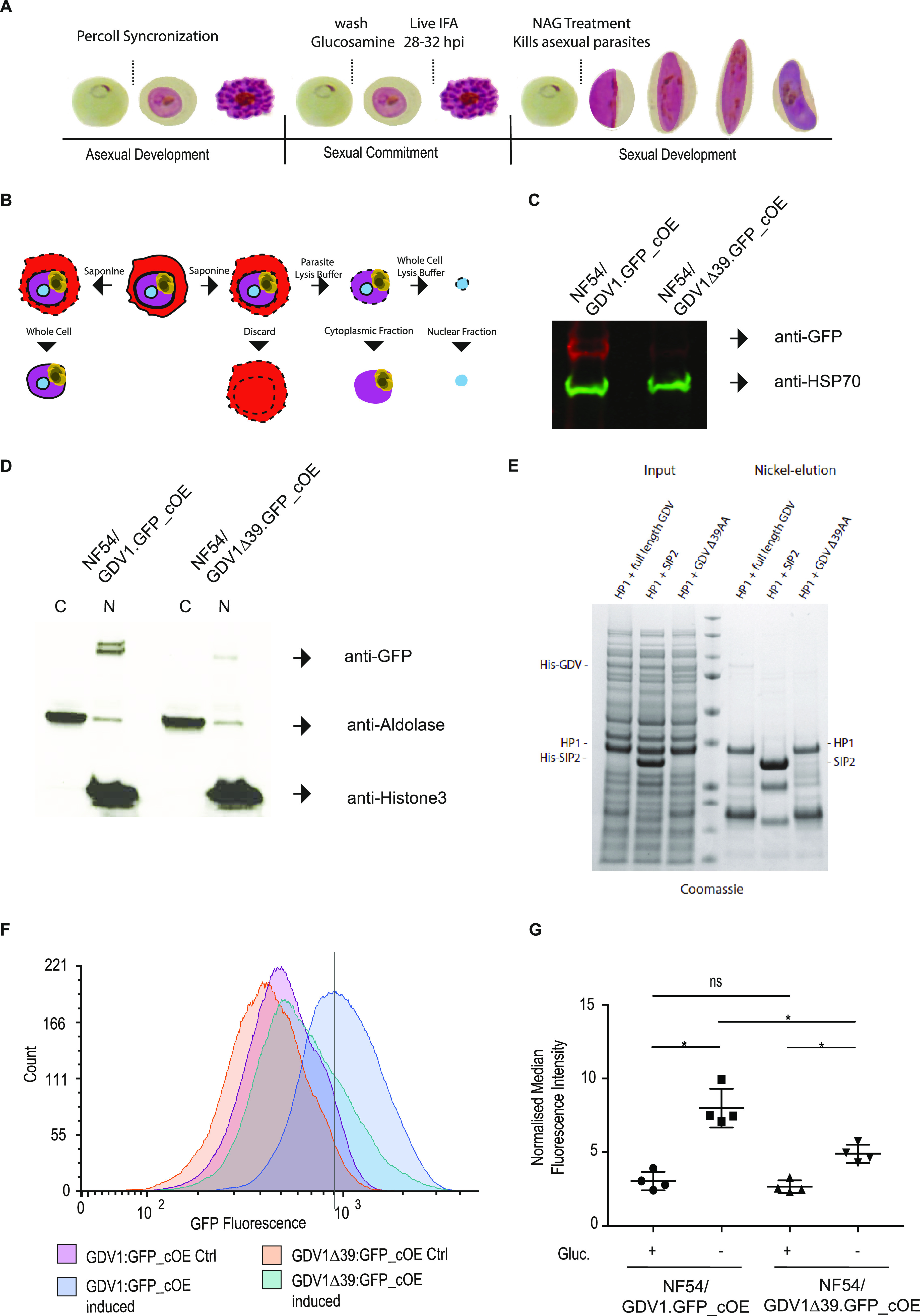
GDV1Δ39 expression, localization, and interaction with HP1. (A) Representation of the protocol used to collect the samples used to characterize expression and localization of GDV1Δ39:GFP. (B) Illustration of the subcellular fractionation workflow. (C) Western blot showing the levels of GDV1:GFP expression in NF54/GDV1:GFP_cOE and GDV1Δ39:GFP in NF54/GDV1Δ39:GFP_cOE parasites grown in the absence of glucosamine (induces expression); GDV1:GFP/GDV1Δ39:GFP expression is detected using an anti-GFP antibody while anti-HSP70 antibodies have been used as controls. (D) Western blot showing GDV1:GFP expression levels in the cytoplasmic and nuclear fraction in NF54/GDV1:GFP_cOE and NF54/GDV1Δ39:GFP_cOE parasites cultured in the absence of glucosamine (induces expression). (E) Strep-HP1 copurifies with both His-GDV1 and His-GDV1Δ39 but not with the His-SIP2 control. Coomassie-stained SDS-polyacrylamide gel from pulldown experiment with His-GDV1/Strep-HP1 and His-SIP2/Strep-HP1. Lane 4: protein size standard. (F) Representative normalized flow cytometry histograms quantifying GDV1:GFP fluorescence for each parasite line. Solid line indicates the position of the peak for the wild-type NF54/GDV1:GFP_cOE line. (G) Quantification of the median fluorescence intensity of GDV1:GFP in induced or uninduced NF54/GDV1:GFP_cOE and NF54/GDV1Δ39:GFP_cOE parasite lines, normalized to uninfected parasites from each experiment, ± SD, *n* = 4. *, *P* < 0.05; ns, not significant, *P* ≥ 0.05; NF54/GDV1:GFP_cOE uninduced versus induced, *P* = 0.0227; NF54/GDV1Δ39:GFP_cOE uninduced versus induced, *P* = 0.0318; NF54/GDV1:GFP_cOE induced versus NF54/GDV1Δ39:GFP_cOE induced *P* = 0.0227; NF54/GDV1:GFP_cOE uninduced versus NF54/GDV1Δ39:GFP_cOE uninduced, *P* = 0.1235. Statistical analysis was performed using Holm-Sidak corrected multiple comparison analysis of variance (ANOVA).

GDV1 is a nuclear protein, and we hypothesized that the deletion of a predicted C-terminal nuclear bipartite localization sequence (cNLS mapper, http://nls-mapper.iab.keio.ac.jp/cgi-bin/NLS_Mapper_form.cgi) may interfere with GDV1 nuclear localization and hence its ability to interact with HP1 at heterochromatic loci. Therefore, we localized induced GDV1:GFP and GDV1Δ39:GFP by immunofluorescence at 28 to 32 hpi (see Fig. 4A for reference). As expected, control GDV1:GFP_cOE parasites showed a clear punctate and nuclear GDV1:GFP signal (Fig. S3E) (12). GDV1Δ39:GFP_cOE also showed localized GDV1Δ39:GFP signal in the nucleus, but the signal was weaker and more diffuse compared with that of GDV1:GFP (Fig. S3F). In order to quantify and compare GFP levels in GDV1:GFP_cOE and GDV1Δ39:GFP_cOE parasites, we performed a whole-cell protein extraction for Western blotting using GFP-specific antibodies. HSP70-specific antibodies were used as a loading control (Fig. 4B and C). The Western blot (WB) showed a clear reduction of GDV1Δ39:GFP compared to GDV1:GFP (Fig. 4C). To quantify the localization of GDV1Δ39:GFP in the cytoplasm compared to that in the nucleus, we prepared cytosolic and nuclear protein extracts using subcellular fractionation (Fig. 4B and D). We determined the cytoplasmic fraction using anti-aldolase antibodies (31), and anti-histone 3 antibodies were used to determine the nuclear fraction (32). GDV1Δ39:GFP was only detected in the nuclear fraction, further supporting that its nuclear localization is not affected by the C-terminal truncation (Fig. 4D). To test if the GDV1Δ39 deletion affects its interaction with HP1, we performed an *in vitro* assay where 6×His-tagged GDV1 (His-GDV1) and GDV1Δ39 (His-GDV1Δ39) versions were coexpressed with Strep-tagged HP1 in Escherichia coli bacteria. Interaction between His-GDV1 and Strep-HP1 is detected by affinity purification of His:GDV1 and analysis of coeluted proteins by Coomassie staining (12). His-tagged SIP2 does not interact with Strep-HP1 and was used as a negative control (Fig. 4E). As previously shown, His-GDV1 pulled down HP1, which was not observed when SIP2 was used as a bait (12). Interestingly, His-GDV1Δ39 also pulled down HP1, showing that the GDV1 C terminus is not essential for the interaction in E. coli (Fig. 4E). This observation indicates that the interaction of GDV1Δ39:GFP and HP1 could still occur in the parasite but that it is insufficient to trigger gametocytogenesis.

An explanation for the lack of induction despite the apparent correct localization could be that GDV1Δ39:GFP levels do not reach the threshold required for efficient gametocyte induction. To examine expression of the GDV1Δ39:GFP, we analyzed protein levels at the single-cell level using flow cytometry in uninduced and induced NF54/GDV1:GFP_cOE and NF54/GDV1Δ39:GFP_cOE parasites (Fig. 4F and G and Fig. S4). As expected, NF54/GDV1:GFP_cOE parasites show a robust increase of GDV1:GFP expression through glucosamine removal. A measurable increase of the mean fluorescence of GDV1Δ39:GFP was also observed upon induction in most NF54/GDV1Δ39:GFP_cOE parasites but at levels well below the GDV1:GFP levels observed for NF54/GDV1:GFP_cOE parasites. The reduction on the protein levels of GDV1Δ39:GFP compared to those of GDV1:GFP is not explained by differences in transcript level. Quantitative reverse transcription-PCR (RT-qPCR) shows ∼1.5-fold higher transcript levels of *gdvΔ39:gfp* compared to those of *gdv1:gfp*, while both lines show an equal increase upon induction (∼2-fold) (Fig. S4D and E). The increase of transcript levels for *gdv1Δ39:gfp* is likely explained by an additional copy of the plasmid that integrated into the genome: qPCR analysis of genomic DNA from NF54/GDV1Δ39:GFP_cOE parasites using primers specific for GFP showed an ∼1.8-fold increase of DNA content compared to that of genomic DNA (gDNA) from NF54/GDV1:GFP_cOE parasites (data not shown). Interestingly, a small proportion of NF54/GDV1Δ39:GFP_cOE parasites displayed GDV1Δ39:GFP fluorescence at a level similar to that of GDV1:GFP in the NF54/GDV1:GFP_cOE control line. In line with its nuclear localization, GDV1Δ39:GFP may therefore contribute to the formation of gametocytes in these parasites.

10.1128/mSphere.01093-20.4 FIG S4 Quantification of transcript and protein levels of GDV1_OE parasite lines. (A) Gating strategy for flow cytometry experiments. (B) Flow cytometry histograms quantifying GFP fluorescence in uninfected RBC and RBC infected with ring stage parasites, trophozoites, and schizonts. Stacked plots are shown for both GDV1:GFP uninduced and induced parasites. (C) Normalized flow cytometry histograms quantifying GFP fluorescence for each line. The experiment was repeated 4 times with similar results. Dotted lines indicate the position of the peaks for the wild-type NF54/GDV1:GFP_cOE line. (D) RT-qPCR quantification of *gdv1_gfp* comparing transcript levels in uninduced and induced NF54/GDV1Δ39_GFP and NF54/GDV1_GFP parasites. Values have been calculated relative to the eukaryotic translation initiation factor 2-alpha kinase (pk4, PF3D7_0628200 and actin). Results are the mean of three biological replicates (error bars represent SD). (E) RT-qPCR quantification of induced/uninduced NF54/GDV1Δ39_GFP and induced/uninduced NF54/GDV1_GFP parasites. Results are the mean of three biological replicates (error bars represent SD). Download FIG S4, PDF file, 1.2 MB. Copyright © 2021 Tibúrcio et al. 2021 Tibúrcio et al. https://creativecommons.org/licenses/by/4.0/ This content is distributed under the terms of the Creative Commons Attribution 4.0 International license.

## DISCUSSION

The aim of this study was to identify nonessential kinases as regulators of gametocyte commitment/development in P. falciparum. While several parasite lines of the kinase knockout collection (14) were able to form gametocytes, two kinase KO lines showed a gametocytogenesis phenotype that led to the identification of a nonsense mutation in *gdv1* that results in a 39-amino-acid (aa) truncation of the GDV1 C terminus. This mutation may have been acquired by the common parental line prior to generation of the original transgenic lines, although several other clones from the Solyakov study that we tested here are able to form gametocytes, possibly reflecting that only a proportion of the parasite population in the parental line carried the mutation. Alternatively, it cannot be excluded that the mutation arose independently in these two lines. Regardless of the origin of the mutations, our results show that the premature stop codon mutation in *gdv1* resulting in a 39-amino-acid C-terminal truncation in the *tlk2* and *elk2* KO lines is sufficient to abolish sexual commitment. Loss of gametocytogenesis is a common occurrence in culture-adapted strains (2), and spontaneous loss-of-function mutations for *gdv1* leading to loss of gametocytogenesis were previously identified (33, 34). Our results extend previous studies of GDV1 truncations: we observed that the truncated GDV1Δ39:GFP protein was present at substantially reduced levels compared to those of full-length GDV1:GFP. We propose that the GDV1Δ39:GFP truncation leads to reduced GDV1 protein stability, which is most likely the underlying cause for the lack of gametocytes in the GDV1Δ39 mutants. However, the truncation of GDV1 results neither in a strong nuclear localization defect when overexpressed as a GFP fusion protein nor in a failure to interact with HP1 expressed in bacteria. It will be important to show in the future whether the few NF54/GDV1Δ39:GFP_cOE parasites, which show similar levels of GDV1Δ39:GFP to those of GDV1:GFP in NF54/GDV1:GFP_cOE parasites, are able to induce gametocytogenesis. If they fail to do so, it could point toward additional functions of the GDV1 C terminus, potentially contributing to bringing GDV1 to the *ap2-g* locus through interactions with a yet unknown protein.

## MATERIALS AND METHODS

### Plasmid construction and transfection.

The construction of each of the ePK knockout plasmids here characterized has been described in reference 14. The *pMK-RQ-tkl2-loxPint* donor plasmid (synthesized by Geneart) contains a recodonized (rc) version of sequence containing the glycine-rich loop in the kinase domain of *tkl2* (rc. Gly loop) flanked by two loxPints and homology regions for homology-directed repair. The *pDC2-Cas9-hDHFRyFCU* guideRNA plasmid targeting *tkl2* locus (pDC2_TKL2_gRNA) was generated using the primer pairs pDC2_TKL2_gRNA1_FOR/pDC2_TKL2_gRNA1_REV. Because we did not have a 3D7::DiCre line, we generated the 3D7/TKL2:loxPint conditional KO line by doing, for the first time, a double transfection with the *pMK-RQ-tkl2-loxPint* and pDC2_TKL2_gRNA, together with the pBSPfs47DiCre (containing the DiCre cassette) and the CRISPR/Cas9 plasmid pDC287 containing the guide RNA targeting the Pfs47 locus, as previously described (23). The plasmids were suspended in 100 μl of P3 primary cell solution, 40 μg of each rescue plasmid, and 20 μg of *pDC2-Cas9-hDHFRyFCU* guide RNA for each respective rescue plasmid and transfected into the 3D7 parasites. Briefly, purified P. falciparum 3D7 schizont stages were electroporated using Amaxa 4D-Nucleofector (Lonza), program FP158 (35). Transfected parasites were selected using 5 nM WR99210 (Jacobus Pharmaceutical), and after a first round of selection, parasites were cloned.

To generate the *pMK-RQ-gdv1Δ39-HA* plasmid, which upon integration into the endogenous *gdv1* locus mimics the mutation found in the kinase KO lines, the *gdv1* (PF3D7_0935400) 3′ homology region was PCR amplified from NF54 genomic DNA with primers 268/269 (Table S2). The amplified PCR fragment was Gibson-cloned into an AfIIII-digested plasmid synthesized by Geneart that contains a *gdv1* 5′ homology sequence followed by a recodonized truncated *gdv1Δ39* version and the sequence encoding the 3×HA tag (Table S2). To generate the pD_cg6_cam-gdv1Δ39-gfp-glmS plasmid, we amplified the gdv1Δ39 sequence from the pMK-RQ-gdv1Δ39-HA plasmid using primers 383/384 (Table S2) and introduced the PCR fragment using Gibson assembly into the donor plasmid pD_cg6_cam-gdv1-gfp-glmS (29) digested with EagI and BsaBI. The guideRNA cassette to mutate endogenous gdv1 was generated using the primer pairs pDC2_GDV1Δ39_gRNA1_FOR/pDC2_GDV1Δ39_gRNA1_REV and cloned into the pDC2-Cas9-hDHFRyFCU plasmid as previously described (22). The rescue plasmid *pMK-RQ-gdv1Δ39-HA* and the CRISPR/Cas9 plasmid *pDC2-Cas9-hDHFRyFCU* were suspended in 100 μl of P3 primary cell solution and 40 μg and 20 μg DNA, respectively, and transfected using Amaxa 4D-Nucleofector (Lonza). Briefly, purified P. falciparum NF54::DiCre schizont stages were electroporated using program FP158 (35). Selection of parasites transfected was done using 5 nM WR99210 (Jacobus Pharmaceutical), and after a first round of selection, parasites were cloned. Transfection of NF54 parasites using the CRISPR/Cas9 pHF_gC-cg6 suicide plasmid (29) and the *pD_cg6_cam-gdv1Δ39-gfp-glmS* donor construct was performed as described previously (12). Fifty μg each of the suicide plasmid and donor plasmid was transfected and parasites were cultured in the presence of glucosamine to block NF54/GDV1Δ39:GFP_cOE protein overexpression. Twenty-four hours after transfection and for six subsequent days in total, the transfected populations were treated with 4 nM WR99210 and then cultured in the absence of drug selection until a stably propagating transgenic population was obtained. All primers, guide RNAs, and fragments used in the construction and integration of the constructs as well as confirmation of rapamycin-mediated excision are described in Table S2.

### *Plasmodium falciparum in vitro* culture of asexual and sexual blood stages.

Plasmodium falciparum parasite lines used in this study were all derived from the NF54 strain (originally isolated from an imported malaria case in the Netherlands in the 1980s [BEI Resources, catalog number MRA-1000]) (36). Asexual parasites were cultured in human blood (UK National Blood Transfusion Service) and RPMI 1640 medium containing 0.5% wt/vol AlbumaxII (Invitrogen) at 37°C, as previously described (37). Asexual parasites were used to produce gametocytes by seeding asexual rings at 1% or 3% parasitemia and 4% hematocrit on day 0 and feeding the parasites once a day during 15 days (day 0 to day 14) in 3% O_2_-5% CO_2_-92% N_2_ gas, in RPMI complemented with 25 mM HEPES, 50 mg/liter hypoxanthine, 2g/liter sodium bicarbonate, 10% human serum (37, 38).

### *Plasmodium falciparum* sexual induction.

An asexual ring stage culture (3%) was induced for sexual conversion using 50% spent medium, expecting the sexually committed merozoites to invade and develop during the next cycle (20, 37). The overexpressing NF54/GDV1:GFP_cOE and NF54/GDV1Δ39:GFP_cOE parasite lines were kept in the constant presence of 2.5 mM glucosamine to block ectopic GDV1 expression and therefore sexual induction, while sexual induction was achieved by culturing the parasites in the absence of glucosamine, as previously described (29).

### Time course of gametocyte induction, RNA extraction, and RNA-seq library preparation.

The samples were collected during the asexual cycle at 28 to 32 hpi and in the matching cycle at 28 to 32 hpi after induction of sexual commitment. The infected RBCs pellets were collected at the respective time point, centrifuged and solubilized in 10 volumes of TRIzol (Ambion) prewarmed to 37°C, lysed for 5 min by mixing vigorously at 37°C, and immediately frozen at –80°C until extraction. Complete RNA was isolated from the samples using TRIzol/chloroform extraction followed by isopropanol precipitation, and its concentration and integrity was verified using Agilent Bioanalyzer (RNA 6000 Nano kit) and NanoDrop 1000 spectrophotometer. One to two μg of total RNA from each sample (or complete sample if the yield was lower) was used for mRNA isolation (magnetic mRNA isolation kit, NEB). First-strand cDNA synthesis was performed using the SuperScript III first-strand synthesis system and a 1:1 mix of Oligo(dT) and random primers (Invitrogen). The DNA-RNA hybrids were purified using Agencourt RNACleanXP beads (Beckman Coulter) and the second cDNA strand was synthetized using a 10 mM dUTP nucleotide mix, DNA polymerase I (Invitrogen), and RNAseH (NEB) for 2.5 h at 16°C. The long cDNA fragments were purified and fragmented using a Covaris S220 system (duty cycles = 20, intensity = 5, cycles/burst = 200, time = 30 s). The ∼200-bp-long fragments were end-repaired, dA-tailed, and ligated to “PCR-free” adapters (39) with index tags using NEBNext according to the manufacturer’s instructions. Excess adapters were removed by two rounds of cleanup with 1 volume of Agencourt AMPure XP beads. Final libraries were eluted in 30 μl water, quality-controlled using Agilent Bioanalyzer (high sensitivity DNA chip), digested with USER enzyme (NEB), and quantified by qPCR. For some libraries, additional 5 cycles of PCR amplification were performed, using KAPA HiFi HotStart PCR mix and Illumina tag-specific primers to obtain enough material for sequencing. Pools of indexed libraries were sequenced using an Illumina HiSeq2500 system (100-bp paired-end reads) according to the manufacturer’s manual. All samples were generated in duplicates or triplicates, and uninduced controls were always generated and processed in parallel. Raw data are available through GEO database repository (study GSE158689).

### RNA-seq data analysis.

The generation of raw data in the form of *.cram files quality control and adapter trimming was performed using the default analysis pipelines of the Sanger Institute. The raw data were transformed into paired *.fastq files using Samtools software (version 1.3.1). The generated reads were realigned to Plasmodium falciparum genome (PlasmoDB-30 release) in a splice aware manner with HISAT2 (40) using –known-splicesite-infile option within the splicing sites file generated based on the current genome annotation. Resulting *.bam files were sorted and indexed using Samtools and inspected visually using Integrated Genome Viewer (version 2.3.91). High-throughput sequencing (HT-seq) python library (40) was used to generate reads counts for all genes for further processing. Raw counts were normalized to median-ratio and then tested against linear models of time nested in line and line nested within time using a negative binomial model for the normalized counts using DESeq2, differential genes being selected for a false-discovery rate of <0.1 (41).

### Saponin lysis and whole-cell, cytoplasmic, and nuclear protein extraction.

Ten ml of parasite culture (2 to 5% parasitemia, 4% hematocrit) was transferred to a 15-ml tube and centrifuged at 600 × *g* for 5 min. The supernatant was aspirated and the RBC pellet was resuspended in 5 volumes of 0.15% saponin solution (2.5 ml for 500 μl RBC). After an incubation on ice of maximum 10 min, the parasites were centrifuged at 1,503 × *g* for 5 min at 4°C. Subsequent steps were performed on ice in order to prevent protein degradation. The supernatant was aspirated and the parasite pellet was resuspended in 1 ml of cold phosphate-buffered saline (PBS) and transferred to an Eppendorf tube. The parasite pellet was centrifuged at 1,503 × *g* for 30 sec at 4°C and washed with cold PBS until the supernatant was clear.

For whole-cell protein extraction, one pellet volume (30 to 50 μl) of whole-cell protein lysis buffer (8 M urea, 5% SDS, 50 mM Bis-Tris, 2 mM EDTA, 25 mM HCl [pH 6.5]) complemented with 1× protease inhibitor cocktail (Merck) and 1 mM DTT was added to the pellet at RT in order to lyse the parasites. The tube was vortexed, heated to 94°C for 5 min, sonicated for 2 min (5 cycles of 30 sec on/30 sec off), vortexed, and heated again. Subsequently, the protein sample was centrifuged at 20,238 × *g* for 5 min at room temperature and the supernatant was transferred into a new tube, which was frozen at −20°C and stored until use.

For cytoplasmic and nuclear protein extraction, the parasite pellet was lysed in 300 μl of cytoplasmic lysis buffer (20 mM HEPES [pH 7.9], 10 mM KCl, 1 mM EDTA, 0.65% Igepal) complemented with 1× protease inhibitor cocktail (Merck) and 1 mM DTT (leaving the nucleus intact) and incubated on ice for 5 min (43). The lysed parasites were centrifuged at 845 × *g* for 3 min, and the supernatant representing the cytoplasmic protein fraction was transferred into a new tube and placed on ice. The remaining nuclear pellet was washed in 500 μl of cytoplasmic lysis buffer and centrifuged at 845 × *g* for 3 min. The washing was repeated until the supernatant was clear. The nuclear pellet was resuspended in 60 μl of whole-cell lysis buffer and vortexed at high speed at room temperature for 10 to 20 min. The insoluble material was centrifuged at 20,238 × *g* for 3 min, and the supernatant representing the nuclear protein fraction was transferred to a new tube and placed on ice. Both protein fractions were frozen at −20°C and stored until use.

### Western blotting.

Parasite extracts were solubilized in protein loading buffer, denatured at 95°C for 10 min, subjected to SDS-PAGE, and transferred onto a nitrocellulose membrane. Membranes were immunostained with mouse anti-GFP (1:250 dilution; Roche, 11814460001), rabbit anti-Aldolase-horseradish peroxidase (HRP) conjugated (1:5,000 dilution; Abcam ab38905), and rabbit anti-histone 3 (1:2,000 dilution; Abcam ab1791) primary antibodies. Antibody detection was done using chemiluminescent Western blotting using goat anti-mouse secondary antibody conjugated with HRP and the ECL Western blotting detection reagents (Amersham RPN2106) or by direct infrared fluorescence detection on the Odyssey Infrared Imaging System (Odyssey CLx, LI-COR) using IRDye 680LT goat anti-rat IgG (1:10,000 dilution; LI-COR) and IRDye 800CW goat anti-rabbit IgG (1:10,000 dilution; LI-COR).

### Immunofluorescence assay at different parasite stages.

Air-dried thin blood films of asexual parasites were fixed with 4% paraformaldehyde containing 0.0075% glutaraldehyde for 15 min and permeabilized in 0.1% (vol/vol) Triton X-100 (Sigma) for 10 min (42). Blocking was performed in 3% bovine serum albumin (BSA) for 1 h. Slides were incubated with rat anti-HA high-affinity (1:1,000 dilution; Roche, clone 3F10) at room temperature for 30 min, followed by Alexa fluor conjugated goat anti-rat IgG (1:1,000 dilution; Thermo Fisher Scientific) at room temperature for 30 min. Parasite nuclei were stained with 4′,6-diamidino-2-phenylindole (DAPI; Invitrogen). Slides were mounted in ProLong Gold antifade reagent (Invitrogen) and images were obtained with the inverted fluorescence microscope (Ti-E; Nikon, Japan) and processed using NIS-Elements software (Nikon, Japan).

### Flow cytometry.

NF54/GDV1:GFP_cOE and NF54/GDV1Δ39:GFP_cOE parasites were grown in the presence or absence of glucosamine in order to block or allow sexual commitment, respectively. Schizonts were purified by Percoll gradient and allowed to invade fresh red blood cells for 4 h before uninvaded schizonts were removed. Flow cytometry analysis was performed at approximately 44 h post invasion, in 4 biological replicates. For one replicate, parasites were fixed for 1 h in 4% paraformaldehyde in PBS, stained with Hoechst 33342 (1:1,000 in PBS) for 10 min, and analyzed on an LSRFortessa flow cytometer (Becton, Dickinson) using FACSDiva software. For the other three replicates, live parasites were stained with Hoeschst 33342 and analyzed on a BD FACSAria II flow cytometer (Becton, Dickinson) using FACSDiva software. Hoechst fluorescence was detected using a 355 nm (UV) excitation laser with a 450/50 nm bandpass filter, while GFP fluorescence was detected with a 488 nm (blue) excitation laser, a 505 nm longpass filter, and a 530/30 nm bandpass filter. At least 30,000 cells were counted for each sample. Data were analyzed using FCS Express 7 (Research Edition) software. The population was first gated on single cells based on the side and forward scatter, then on highly Hoechst-positive infected schizonts, before the median fluorescence intensity (MFI) of the GFP fluorescence was calculated for each line. An example of the gating strategy for infected cells is shown in Fig. S4. Due to the variation in fluorescence intensity between different experiments, MFI values were normalized by dividing the MFI of each infected sample by the average MFI of the uninfected samples within the same experiment (*n* = 4). Statistical analysis was performed using Holm-Sidak corrected multiple comparison analysis of variance (ANOVA) on samples paired within each experiment using GraphPad Prism version 8.

### Reverse transcription quantitative PCR.

NF54/GDV1:GFP_cOE and NF54/GDV1Δ39:GFP_cOE parasites were synchronized to a 4-h window and grown in the presence or absence of glucosamine in order to block or allow sexual commitment, respectively. Schizonts from the 3 independent biological replicates for fluorescence-activated cell sorter (FACS) analysis were harvested at 40 to 44 hpi and used for RNA extraction. The infected RBCs pellets were solubilized in 10 volumes of TRIzol (Ambion) prewarmed to 37°C, lysed for 5 min by mixing vigorously at 37°C, and immediately frozen at −80°C until extraction. Complete RNA was isolated from the samples using TRIzol/chloroform extraction purified using the RNeasy Plus minikit (Qiagen). Residual gDNA was digested with TURBO DNA-free DNase I (Ambion). Three to five μg of total RNA from each sample was reverse-transcribed using the SuperScript III first-strand synthesis system (Invitrogen). qPCRs were performed using KAPA SYBR Fast ROX Low kit (Sigma-Aldrich) in a reaction volume of 20 μl. All reactions were run in technical triplicate. Cycling conditions were 95°C for 3 min, followed by 40 cycles of 95°C/3 sec and 60°C/40 sec. Product-specific amplification was ensured by melting curve analysis for each reaction. Relative transcript levels were calculated by normalization against the housekeeping gene encoding eukaryotic translation initiation factor 2-alpha kinase (*pk4*, PF3D7_0628200). For gDNA generation, compound 2 arrested schizonts where harvested and genomic DNA was extracted using the Qiagen blood and tissue kit, according to the manufacturer’s recommendation. All primer sequences are listed in Table S2.

### *In vitro* protein-protein interaction experiments.

In order to coexpress Strep(II)-tagged HP1 with a His-SUMO-tagged truncated version of GDV1, we deleted the 39 C-terminal amino acids of the coding sequence of GDV1 in the vector pStrep-HP1_HS-GDV1 (12). For this purpose, we circularized a PCR product amplified from this vector with the primers D39F and D39R using Gibson assembly. The proteins were expressed and the *in vitro* interaction assay was performed as previously described (14) using full-length GDV1 as the positive and SIP2 as the negative control.
