# Supplementary figures and images for: A 39-Amino-Acid C-Terminal Truncation of GDV1 Disrupts Sexual Commitment in Plasmodium falciparum
Source: mSphere. 2021 May 19;6(3):e01093-20. doi: 10.1128/mSphere.01093-20 (PMC8265674; doi:10.1128/mSphere.01093-20)

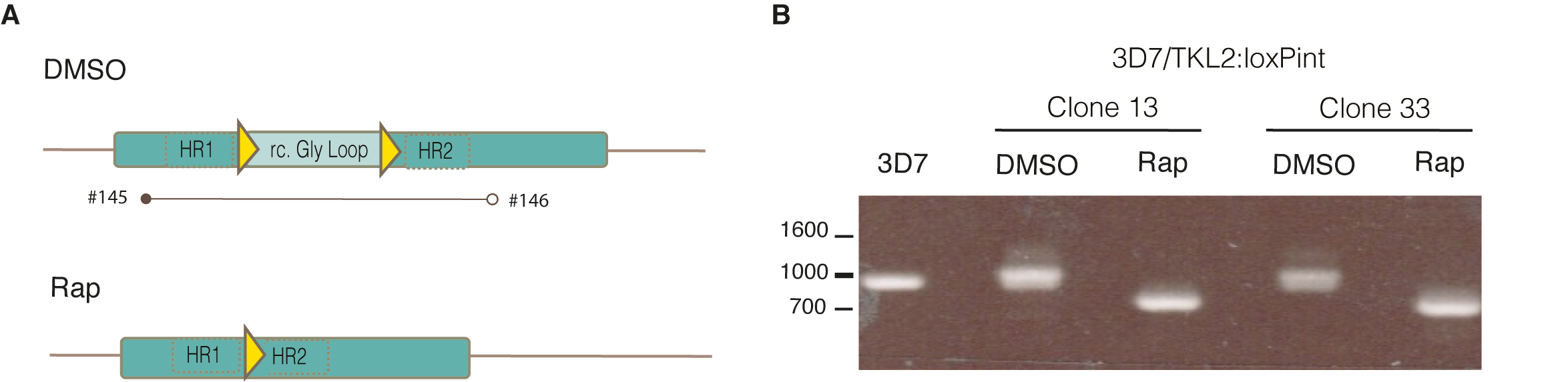

Supplement: FIG S1 [file msphere.01093-20-sf001.jpg]

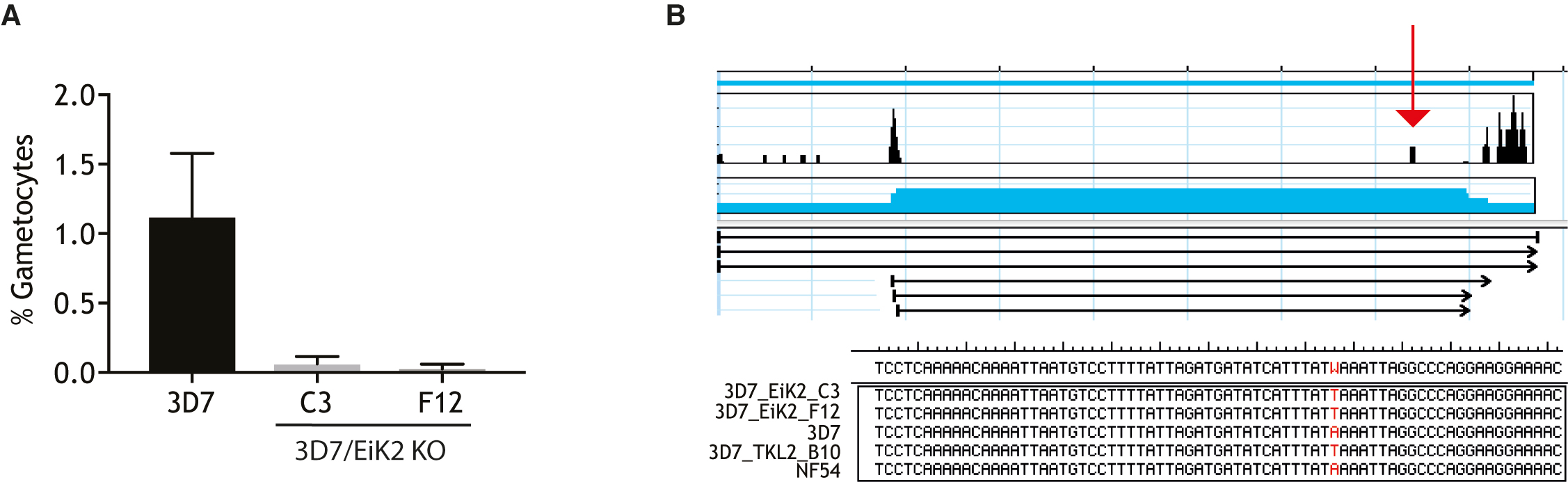

Supplement: FIG S2 [file msphere.01093-20-sf002.jpg]

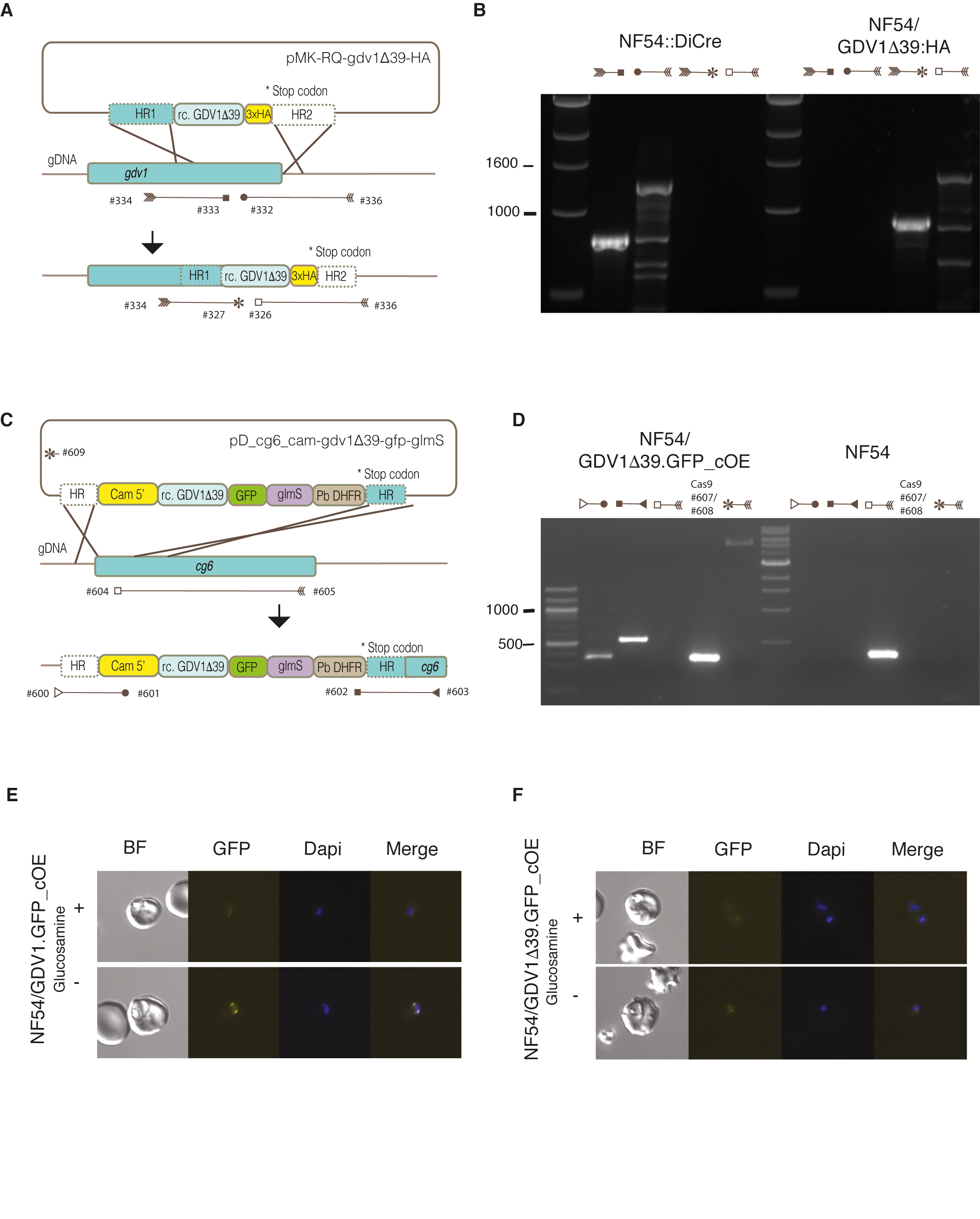

Supplement: FIG S3 [file msphere.01093-20-sf003.jpg]

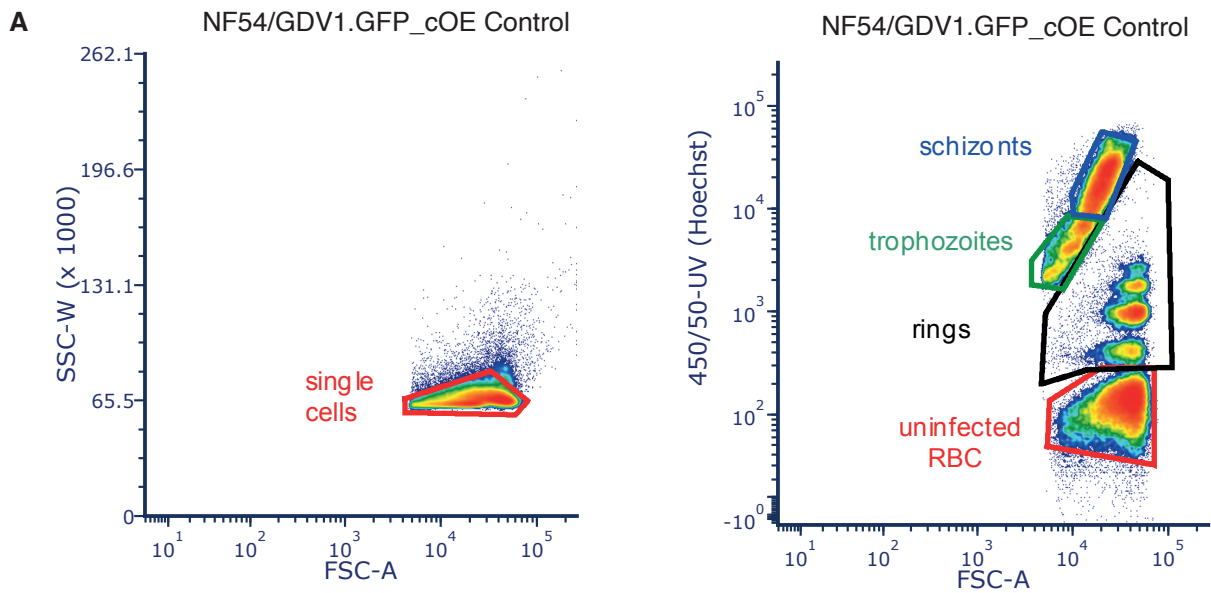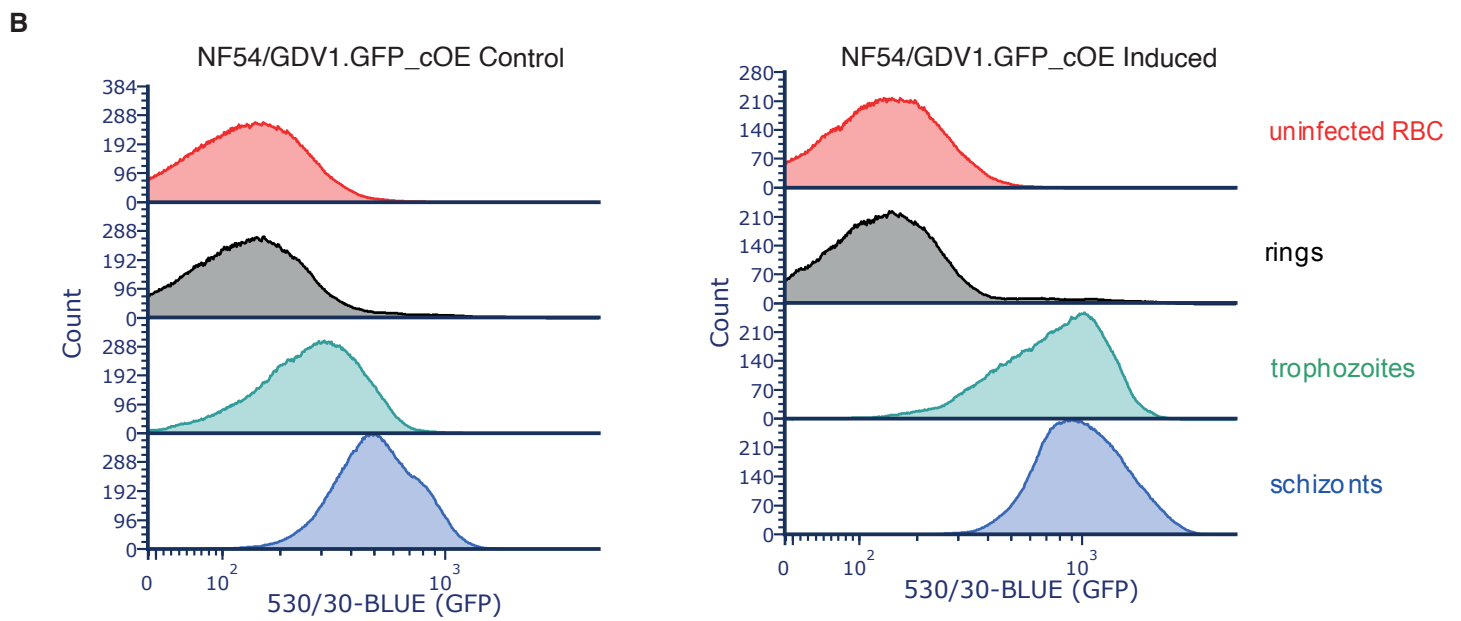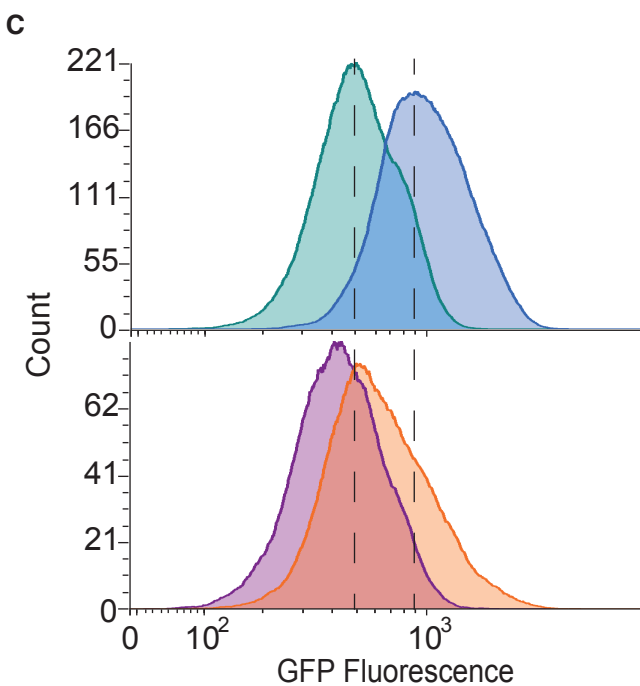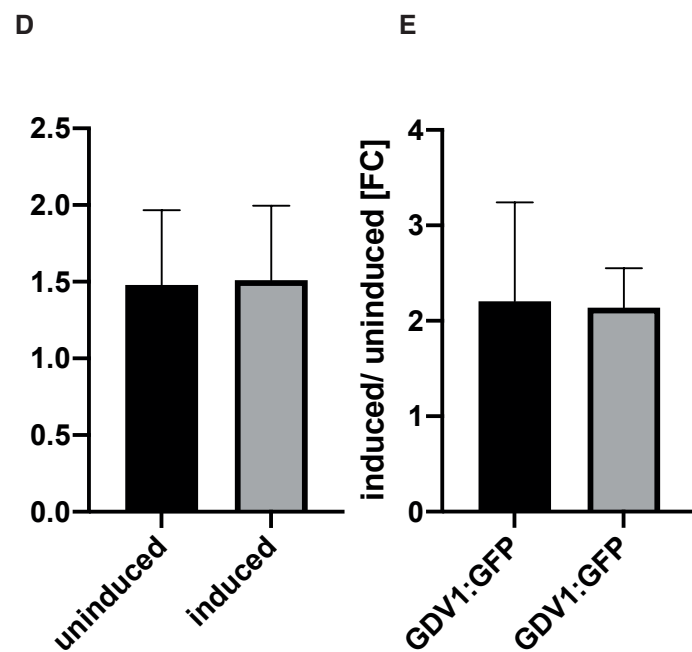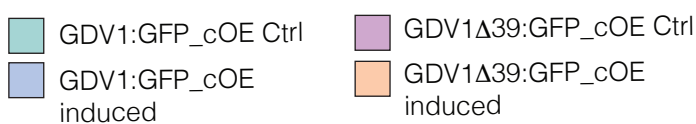

Supplement: FIG S4 [file msphere.01093-20-sf004.pdf]
