# Supplementary material for: A 39-Amino-Acid C-Terminal Truncation of GDV1 Disrupts Sexual Commitment in Plasmodium falciparum
Source: mSphere. 2021 May 19;6(3):e01093-20. doi: 10.1128/mSphere.01093-20 (PMC8265674; doi:10.1128/mSphere.01093-20)
Supplement: TABLE S2 [file msphere.01093-20-st002.docx]

| **Primer** | **Sequence** |
| --- | --- |
| #145 | AGCAAAAGTCTTAAGCTCATGGAGG |
| #146 | ATGAATAGCAGGAGATTTAGATACC |
| pDC2_TKL2_gRNA_FOR | attgATAATAGAATTGCAAAAGGA |
| pDC2_TKL2_gRNA_REV | aaacTCCTTTTGCAATTCTATTAT |
| *tkl2-loxPint* | CTGGAAAGCGGGCAGTGAAAGGAAGGCCCATGAGGCCCAGATAATAGGGGTCTGCATATCCTGGGGTTCCTCCTGTCAAGTTAAACACATTATTGTTGTTCATGTAAACAAAGGAAAGCCCAAAATCACCAAGCTTAGCATTAAATTGATCATCTATTAAAATGTTTGCAGATTTTAAATCTCTATGATATACAATAGGAGAAGATGTATGTAAATAACATAAGACATTAATTATTTGTACTAATATATTTATTCTTATATTAAATGATAAAAATAGAGGTGTGTTATTATCAAATGAATTTTGTTGAAATGGAGAATATTTTGAATTATTGAAATATGGGAAAATGTTTTGTTTATAAAACGTTGAAGTATAATAATTTGTAGAAGAACAAGAAGAAGAACTTTTTTTTTTACGTAGATAATTTTCATAACAACTTATATTATAATAACTTAATTCTGGATTTTCTTTGTTTTTTGAATTATAATAATAATAATGATTAAATAATAATGTTCTTAAATCACCTAGATTAACATATTCATATATTAAATAAAAATTATTTTTATTCGTAGCATAACCTAATAAAGATCTACAAAAAAGAATAAAAATAAAAAATAAAAAATAAAAAAAATAAACATATATATATATATACATATATATACATATATATATCCATATATACATATTAATTCATTTCCATAACTTCGTATAATGTATGCTATACGAAGTTATAACTTGCATATATTTTATACATATTCTGTATTACAAGATGTTGTTGTGTCTGTATCTGGACATGATAATGATTTCGTTTTCGAAACCGTTGTTTTCGTTCTTTTTCAaGACCTTAATGGCAACGTTGATGCAGTTCTTCAAAACaCCTTTGTAAACAGTACCGTTACCACCCTTGGCGATTCCTAAAAGAATATAAAATATATAAATATATATATATATACATATATAATAACTTCGTATAATGTATGCTATACGAAGTTATTGTATATTATTTTTTTTATTTACTATTATATTCTGAAAAATTATTTGTTGCTTCTACTAAATCATTAAAATCATATTTATTAGATAAAATCTTTAAAGGAATAAATATATTCTCTTCACGTTTTTCTATTCTGTTAAATTCCATACCTTCATTTGGTGTTCTTGATTCGACATTTTCTCTATTTATTTCATCTTCATAAATCTCATTTTCTTCATCATACATATTTAAAATGTATTTATCATAAGTATCATATGTTTCTATATATACTACCTTTCTATTCTTATGTCTATTCGAATTCTTTCTATTAATATCGTTGTTATTTTTGATATGATTATTATTTATATGGTTATTGCTCACATGAATATTATTAATATGGTTATTGCTCACATGGATATTATTAATATGGTCATtGKTCACATGGATATTTTTAATATGtCTATTATTCATATAATTGTTTATAtATTTATTATTAATATGTTTATCATTCACATGATGATTATTAATATTGAAATTATGGTTATTATTTATATCAATCTCTTTCTTCTTTTCATCCCCTTCC |
| #268 (GDV1_TAA_GIB FOR1) | TAG GCC CAG GAA GGA AAA CAA AAA CGT CAG CAT TCA TCA TC |
| #269 (GDV1_TAA_GIB REV1) | AGT GAA AGG AAG GCC CAT GAG GCC CAG TGA ACA TGC TAT ATT CTT ATG TAT GTA CC |
| #383 (GDV1_TAA_GIB FOR2) | TGTTCCTAATATAAACTCAGATATAAATGTTGATACC |
| #384 (GDV1_TAA_GIB REV2) | GTTCTTCTCCTTTACTCATGCGGCCATAGATAATATCGTCCAGCAGCAGAAC |
| pDC2_GDV1Δ39 gRNA1_FOR | ATTGCTACAAATTTTGTAATTAAT |
| pDC2_GDV1Δ39_gRNA1_REV | AAACATTAATTACAAAATTTGTAG |
| *gdv1Δ39-HA* | GATTCCGATGTAATATCTTATAGTTATCATTTCGACCATACAATCATTTATTGTATTAAAGATAAATATAAAAATGTTACAAATGTTCCTAATATAAACTCAGATATAAATGTTGATACCATAACACATCCTGAAACAACAAACAACATATCCAATCATTATAAAAATCATAACACTATTATGTTTAAAAAACCTGTGATTCTTATCTGTTCTTTGAATAATTTACCCTTAAAAAATAATAAAATACATGATGATATAATTCTTGACGATTTCTGTTATATGACCTACATTATTAAAACTTTGTATTTTCGTTTTCAATCAAGAAGTATACGCAACATGCTCAAAAGCAATCTGAATAACACCACCAACTTTGTGATTAACCGTATCACCCAGATGAATAATACCAATCGCCTGCTGAAAAACAAGATTAATGTTCTGCTGCTGGACGATATTATCTATTACCCATACGATGTTCCAGATTACGCTTATCCGTATGATGTGCCGGATTATGCGTACCCATACGATGTTCCAGATTACGCTTAAATTAGGCCCAGGAAGGAAAACAAAAACGTCAGCATTCATCATCTTCTACAAAAGGCTTATTCTCCATTCTATTATCCAATTCATTATTATATAAAAGAAAAATGTACATATAAATAAATAAATGAAAAAAAAAAAAAAGAAAAATAAAAAAAAAAAAAAAAAAAAAAAAAAAAGAAAAAAGAAAAAAGAAAAAAGTAAAAAGAAAAAAGAAAAAATTTATATGTAAATATAATTAAAAATTTGTATGCTAATATATGTGTATAATATGTATCTCATCGTGTACATGTATATAATATATATATATTTTGTAGGTAAATATTATATCTACTTTTAACATACATATATATGTAATATATATATATATATATTACTAAAAAATATAAAGGTTTCAAAACATATATGTTTGCTTTTATAATTTTAAAAAAAAATTCTTATCTATGTTGGTACATACATAAGAATATAGCATGTTCA |
| #123 | AAAGGTGTTTTGAAGAACTGCATCAACG |
| #124 | TATCTGGACATGATAATGATTTCG |
| #145 | AGCAAAAGTCTTAAGCTCATGGAGG |
| #146 | ATGAATAGCAGGAGATTTAGATACC |
| #147 | GGAGGGAATGGAACAGTATATAAA |
| #148 | TTTATATACTGTTCCATTCCCTCC |
| #332 | ACAATACTACAAATTTTGTAATTAATCGG |
| #333 | ACAAAATTTGTAGTATTGTTGAGGTTAC |
| #334 | AAGGATATTAATAATCATAGAAAACG |
| #336 | TCAATTAAAATATACAGAACAAGTATCC |
| #402 | AAGAGGTAGAGTTCAATTCATCAAACC |
| #403 | ATCTTTAATTTTATTTTGGTCATGC |
| #404 | AAGGCTTTTTCCATTTTCAAGTGTTCAGG |
| #406 | ACATTGAAGATGGAAGCGTTCAACTAGC |
| #600 (CG6 integration rv) | ATTATGGGAAAATAATCCTTAC |
| #601 (Cam rv) | AGAAGCTCAGAGGCATGC |
| #602 (CG6 integration fw) | CTTTAATTTTATTTTGGTCATG |
| #603 (PbDT 3' For) | GGGAAGGTGTTGCTCAAATAGTG |
| #604 (CG6 WT fw) | GTTCATGCTCCTCAACAAAG |
| #606 (CG6 WT rv) | GAACAAATACATAAGAGCGC |
| #607 (Armin 73) | GCTCAATTCTTTATGTCCACAAC |
| #608 (Armin 124) | CATGTTTTGTAATTTATGGGATAGCG |
| #609 (Amp ORI seq fw) | GCGAGGAAGCGGAAGAGC |
| qPCR_GFP_Rev | TGTTTCATATGATCTGGGTATCTCGCA |
| qPCR_GFP_For | AGTGGAGAGGGTGAAGGTGATGC |
| qPCR_actin_for | AGCAGCAGGAATCCACACA |
| qPCR_actin_rev | TGATGGTGCAAGGGTTGTAA |
| qPCR_pk4_for | CTCATATTCCATACGATGCTCCAT |
| qPCR_pk4_rev | TAAACTGAACCAAATCCTCCCTGT |
